# Elevated neutrophil extracellular traps in systemic sclerosis-associated vasculopathy and suppression by a synthetic prostacyclin analog

**DOI:** 10.1186/s13075-024-03379-6

**Published:** 2024-07-25

**Authors:** Neda Kortam, Wenying Liang, Claire Shiple, Suiyuan Huang, Rosemary Gedert, James St. Clair, Cyrus Sarosh, Caroline Foster, Pei-Suen Tsou, John Varga, Jason S. Knight, Dinesh Khanna, Ramadan A. Ali

**Affiliations:** Division of Rheumatology, Department of Internal Medicine, University of Michigan, 1150 W Medical Center Drive, Ann Arbor, MI 48109 USA

**Keywords:** Systemic sclerosis, Vascular complications, Neutrophil extracellular traps (NETs), Prostacyclin

## Abstract

**Objectives:**

Neutrophils and neutrophil extracellular traps (NETs) contribute to the vascular complications of multiple diseases, but their role in systemic sclerosis (SSc) is understudied. We sought to test the hypothesis that NETs are implicated in SSc vasculopathy and that treatment with prostacyclin analogs may ameliorate SSc vasculopathy not only through vasodilation but also by inhibiting NET release.

**Methods:**

Blood from 125 patients with SSc (87 diffuse cutaneous SSc and 38 limited cutaneous SSc) was collected at a single academic medical center. Vascular complications such as digital ulcers, pulmonary artery hypertension, and scleroderma renal crisis were recorded. The association between circulating NETs and vascular complications was determined using in vitro and ex vivo assays. The impact of the synthetic prostacyclin analog epoprostenol on NET release was determined.

**Results:**

Neutrophil activation and NET release were elevated in patients with SSc-associated vascular complications compared to matched patients without vascular complications. Neutrophil activation and NETs positively correlated with soluble E-selectin and VCAM-1, circulating markers of vascular injury. Treatment of patients with digital ischemia with a synthetic prostacyclin analog boosted neutrophil cyclic AMP, which was associated with the blunting of NET release and reduced NETs in circulation.

**Conclusion:**

Our study demonstrates an association between NETs and vascular complications in SSc. We also identified the potential for an additional therapeutic benefit of synthetic prostacyclin analogs, namely to reduce neutrophil hyperactivity and NET release in SSc patients.

**Supplementary Information:**

The online version contains supplementary material available at 10.1186/s13075-024-03379-6.

## Key message

**•** Neutrophils and NETs are understudied in the pathogenesis of SSc.
**•** Plasma NETs are associated with SSc vascular complications and may identify patients deserving proactive treatments.
**•** Prostacyclin analogs potentially protect against SSc vasculopathy-related NET release by boosting intracellular cyclic AMP.

**Supplementary Information:**

The online version contains supplementary material available at 10.1186/s13075-024-03379-6.

## Introduction

Systemic sclerosis (SSc) is a chronic disease characterized by vascular damage, immune dysregulation, and fibrosis. Microvascular injury is an early event in SSc and occurs in virtually all organs, marking the initiation of SSc pathogenesis and triggering a self-fueling process that ends in tissue fibrosis that disrupts the architecture of the affected tissues [1–3]. Prominent vascular complications of SSc include Raynaud’s phenomenon (the first clinical symptom in over 90% of patients), digital ulcerations, pulmonary artery hypertension (PAH), and scleroderma renal crisis, among others [4]. These vascular complications can be debilitating and life-threatening and generally lack effective treatment. To date, the involvement of endothelial cells, fibroblasts, myeloid cells, and lymphocytes in SSc pathogenesis has been intensely investigated. While neutrophils and their thrombo-inflammatory byproducts called neutrophil extracellular traps (**NETs**) are implicated in diverse vasculopathies ranging from large-vessel in situ thrombosis [5] to severe COVID-19 [6], their potential role in SSc has remained largely unexplored.

NETs are tangles of chromatin and microbicidal proteins that are released from neutrophils in response to infectious and sterile stimuli [7–9]. NET formation (NETosis) has received increasing attention as an amplifier of inflammation and thrombosis in autoimmune and inflammatory diseases such as lupus [10, 11], antiphospholipid syndrome (APS) [11–13], diabetes [14, 15], and—most recently—COVID-19 [6]. Mechanistically, NETs trigger type I interferon production [16, 17]; promote autoantibody formation against nucleus- and granule-derived factors [18, 19]; potentiate endothelial activation and damage [20]; and provoke thrombosis in vascular beds of all sizes [5, 21]. In light of the prominent roles of neutrophils and NETs in the pathogenesis of various autoimmune and inflammatory diseases, we sought here to investigate the roles of neutrophils and NETs in the pathogenesis of SSc, and specifically its vascular complications.

Prostacyclin (PGI_2_) is a member of the prostaglandin family of lipid mediators and an important product of arachidonic acid metabolism [22]. Through inhibition of vascular smooth muscle contraction and platelet aggregation, prostacyclin and its synthetic analogs function as potent vasodilators while also possessing additional antithrombotic and anti-inflammatory properties [23]. Stable synthetic prostacyclin analogs are used in the treatment of a variety of vascular diseases [24]. For example, epoprostenol is approved for the treatment of PAH [25]. Furthermore, iloprost has been shown to improve the severity of Raynaud’s phenomenon and promote the healing of ischemic digital ulcers in SSc [26]. Pharmacologically, prostacyclin analogs activate prostacyclin Gαs-coupled receptors, which stimulate adenylate cyclase to produce the second messenger cyclic adenosine monophosphate (cAMP) leading to increased activity of protein kinase A and thereby supporting relaxation of vascular smooth muscle [27]. We recently found that boosting neutrophil cAMP levels through other mechanisms (such as adenosine receptor agonism and phosphodiesterase inhibition) modulates NET release in the context of diseases such as lupus and APS [28, 29].

To this end, we sought to investigate the hypothesis that neutrophils and NETs are primordial and targetable drivers of SSc vasculopathy and that prostacyclin analogs might be a potential approach to ameliorate SSc vasculopathy not only through vasodilation but also via inhibition of NET release.

## Materials and methods

### Patients and ethical statement

This study complied with all relevant ethical regulations and was approved by the University of Michigan Institutional Review Board. All patients who participated in this study met the 2013 ACR/EULAR criteria for SSc [30] and were recruited through the University of Michigan Scleroderma Program. Informed written consent was obtained from all participants (SSc patients and healthy controls) prior to their enrollment in the study. Patients with diffuse cutaneous SSc or limited cutaneous SSc were defined as having vascular complications if they had a history of at least one of digital ulcers, PAH, or scleroderma renal crisis. A cohort of patients without these manifestations was also assembled. For prostacyclin treatment, four SSc patients with digital ischemia were admitted for epoprostenol infusion (0.45 mg in 150 ml 0.9% sodium chloride administered continuously over 30 h) (Fig. 5A). Blood was collected the week before the infusion and 15 h after the start of the infusion, and neutrophils were isolated to determine neutrophil cAMP content and ex vivo spontaneous NET release, as well as NET release in response to lipopolysaccharides (LPS). For all samples, blood was collected into lavender K2EDTA collection tubes by a trained hospital phlebotomist, and plasma was isolated and stored immediately at -80 °C until the time of testing. Plasma samples were used to test for neutrophil activation marker calprotectin (S100A8/A9), the NET marker MPO-DNA complexes, and the vascular injury markers E-selectin and VCAM-1.

### Quantification of S100A8/A9 (calprotectin)

Calprotectin was measured with the Human S100A8/S100A9 Heterodimer DuoSet ELISA (DY8226-05, R&D Systems) according to the manufacturer’s instructions.

### Quantification of MPO-DNA complexes

MPO-DNA complexes were quantified similarly as previously described [29]. We used several reagents from the Cell Death Detection ELISA kit (Roche) for this protocol. First, a high-binding EIA/RIA 96-well plate (Costar) was coated overnight at 4 °C with anti-human MPO antibody (Bio-Rad 0400-0002), diluted to a concentration of 0.5 µg/mL in coating buffer (Cell Death kit). Next, the plate was washed 3 times with wash buffer (0.05% Tween 20 in PBS) and then blocked with 1% BSA in PBS for 1 h at room temperature. Next, the plate was washed 3 times before incubating for 1 h at room temperature with 1:100 plasma in the blocking buffer. Next, the plate was washed 5 times and then incubated for 1 h at room temperature with 1× anti-DNA antibody (HRP conjugated; Cell Death kit) diluted 1:100 in blocking buffer. After 5 more washes, the plate was developed with 3,3′,5,5′-TMB substrate followed by a 2 N sulfuric acid stop solution. Absorbance was measured at a wavelength of 450 nm with a Synergy HT Multi-Mode Microplate Reader (BioTek).

### Quantification of E-selectin and VCAM-1

Kits for quantitative detection of human E-selectin (DY724) and VCAM-1 (DY809-05) were purchased from Invitrogen and performed according to the manufacturer’s instructions.

### Measurement of intracellular cAMP

cAMP levels in neutrophils from SSc patients were measured using the Bridge-It cAMP Designer fluorescence assay kit (Mediomics, catalog 122,934). Briefly, neutrophils (1 × 10^5^) were washed twice with PBS and resuspended in 100 µL Krebs-Ringer bicarbonate buffer. Samples were centrifuged at 12,000*g* for 2 min at room temperature, and supernatants were discarded. The cAMP designer assay solution was then added to the cell pellet and carefully transferred to a 96-well, black-side, clear-bottom plate. The plate was incubated at room temperature for 30 min before measuring fluorescence with a Synergy HT Multi-Mode Microplate Reader at excitation 480 nm with bandpass 20 and emission 540 nm with bandpass 40.

### Human neutrophil purification and NETosis assays

Blood from SSc patients or healthy volunteers was collected by standard phlebotomy techniques. The blood was then fractionated by density-gradient centrifugation using Ficoll-Paque Plus (GE Healthcare, now Cytiva). Neutrophils were further purified by dextran sedimentation of the red blood cell layer before lysing residual red blood cells with 0.2% sodium chloride. To assess NETosis, neutrophils were resuspended in RPMI medium (Gibco) supplemented with 0.5% bovine serum albumin (BSA, MilliporeSigma) and 0.5% fetal bovine serum (Gibco), which had been heat-inactivated at 56 °C. Neutrophils (1 × 10^5^/well) were cultured in 96-well plates at 37 °C in the presence or absence of lipopolysaccharide (LPS). NETosis was assessed by a SYTOX Green–based assay (Thermo Fisher Scientific), which detects extracellular DNA. After 3 h of culture, SYTOX Green was added to a final concentration of 0.2 µM and incubated for an additional 10 min. Fluorescence was quantified at excitation and emission wavelengths of 485 nm and 520 nm, respectively, using a Cytation 5 Cell Imaging Multi-Mode Reader (BioTek) with the following settings: Light Source: Xenon Flash; Lamp Energy: High; Extended Dynamic Range Read Speed: Normal; Delay: 10 ms; Measurements/Data Point: 10; Read Height: 7 mm.

### Statistical analysis

Groups were compared by t-test and correlations were determined by Pearson’s method. Mean is presented and P values < 0.05 were considered statistically significant.

## Results

### Participant characteristics

Sex, age, and disease subgroups; prevalence of anti-centromere, anti-topoisomerase, and anti-RNA polymerase III autoantibodies; and key clinical characteristics for 125 patients with SSc are detailed in Table 1. Patients who participated in this study met the 2013 ACR/EULAR criteria for SSc [30], and were classified as having vascular complications if they had a history of at least one of digital ulcer, PAH, or scleroderma renal crisis. A cohort of patients without these manifestations was also assembled, with no significant differences observed between the two groups regarding age, sex, and disease duration (Table 1). Age- and sex-matched healthy controls were also included.

**Table 1:**
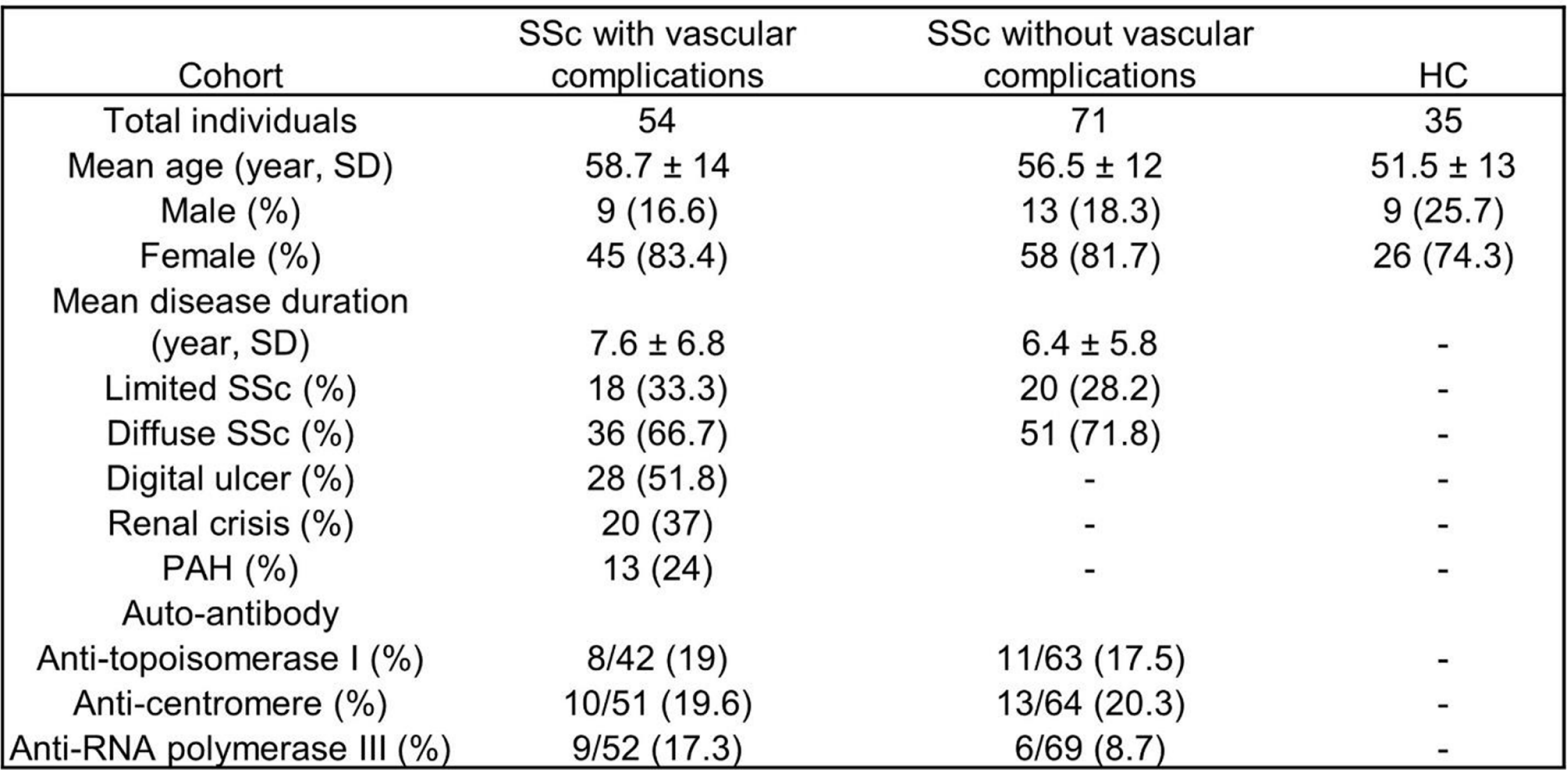
Main features and classifications of SSc patients as well as demographic data for healthy controls (HC). Data are presented as percentages for categorical variables and mean ± standard deviation for continuous variables

### Increased neutrophil activation and NETs in the circulation of SSc patients according to SSc subtype and disease duration

**Fig. 1:**
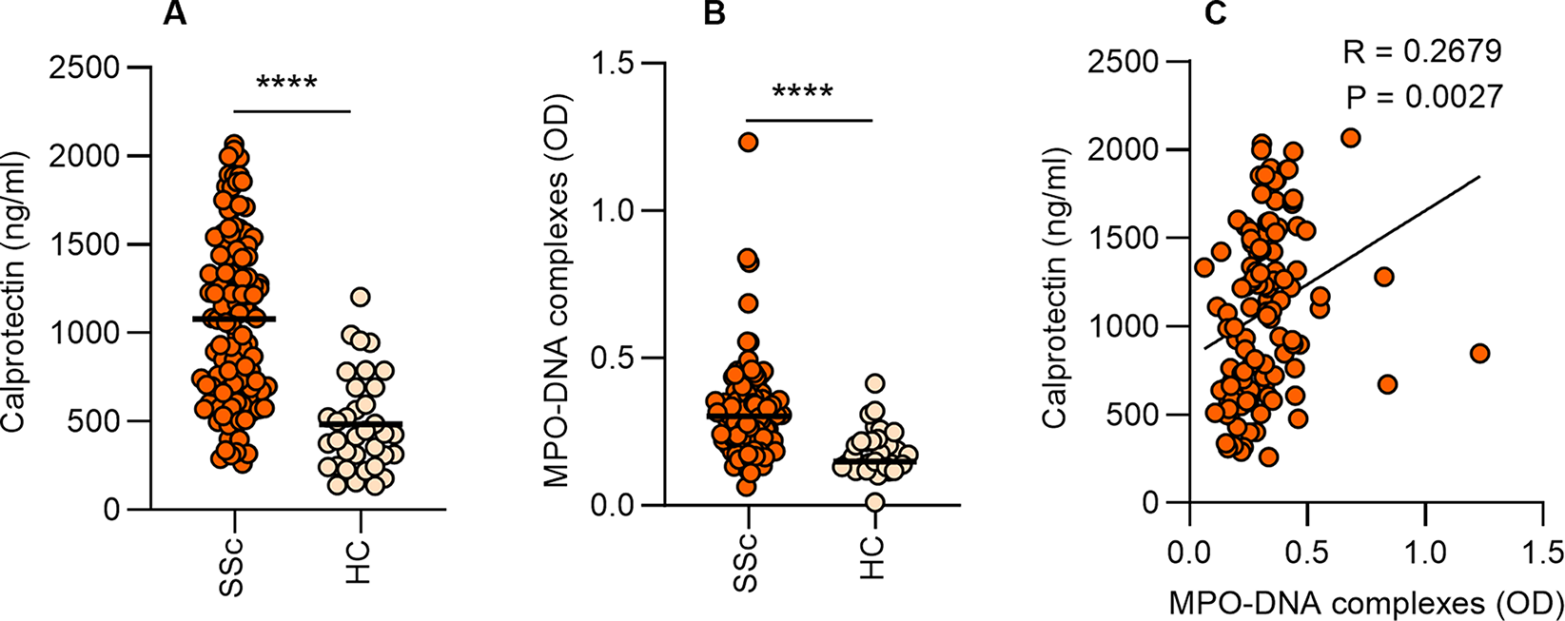
Elevated neutrophil activation and NETs in SSc patients. (**A**-**B**) Levels of calprotectin (S100A8/A9) and MPO-DNA complexes were analyzed by ELISA in plasma samples of SSc patients (*n* = 125) or healthy controls (HC, *n* = 35). (**C**) Correlation between calprotectin (S100A8/A9) and MPO-DNA complexes. Mean is presented as a horizontal line; ****P < 0.0001 by t-test, and Pearson correlation was computed for C

**Fig. 2:**
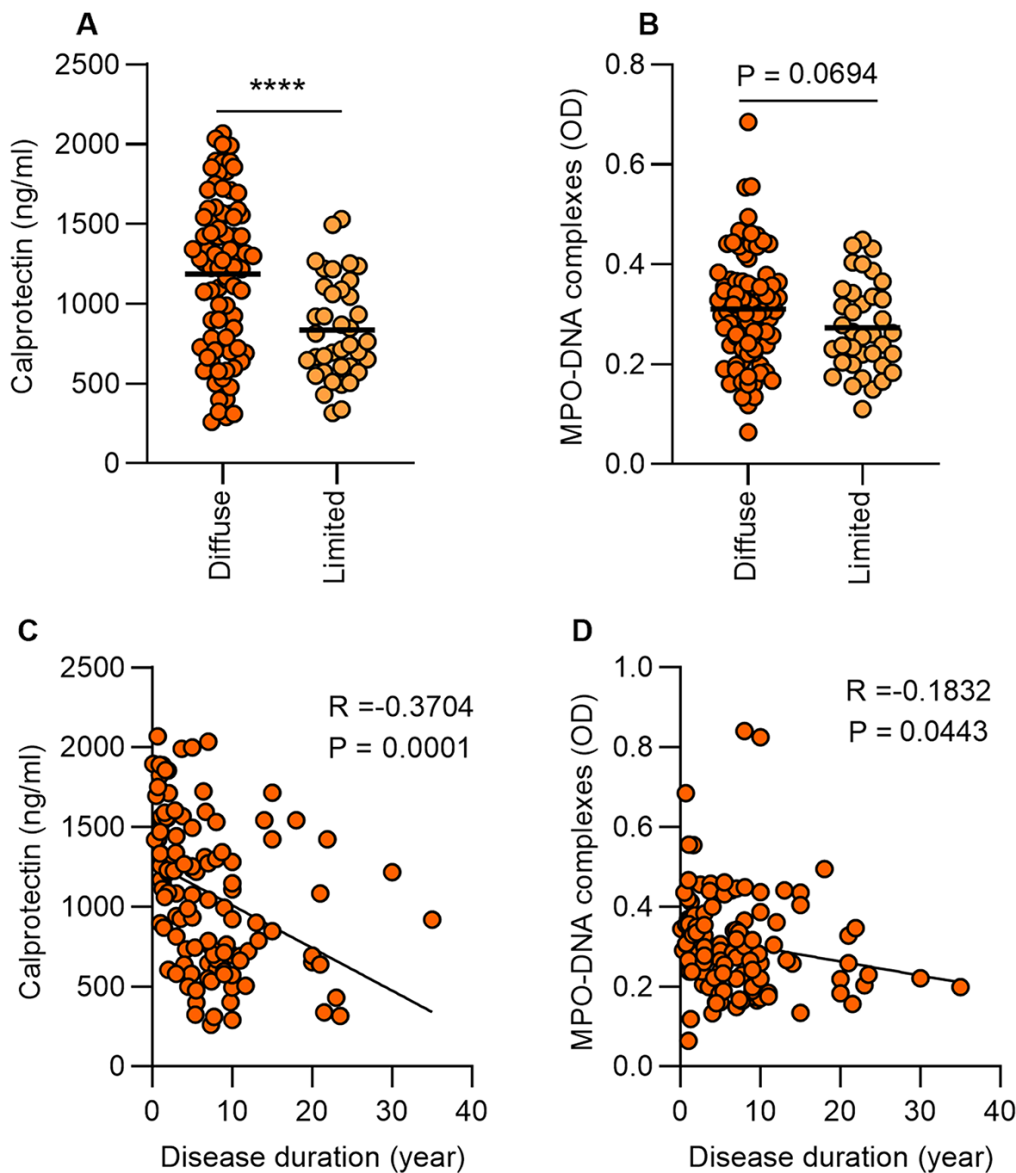
Increased neutrophil activation and NETs in diffuse cutaneous SSc patients and associate with early disease duration. (**A-B**) Levels of calprotectin (S100A8/A9) and MPO-DNA complexes were analyzed by ELISA in plasma samples of SSc patients according to their skin phenotype, diffuse cutaneous SSc (*n* = 87) vs. limited cutaneous SSc (*n* = 38). (**C**) Correlation between neutrophil activation and disease duration. (**D**) Correlation between NET levels in plasma and disease duration. Mean is presented as a horizontal line; ****P < 0.0001 by t-test, and Pearson correlation was computed for C and D

We analyzed plasma and found increased calprotectin (S100A8/A9, a marker of neutrophil activation) and NETs (measured as MPO-DNA complexes) in SSc patients as compared to healthy controls (Fig. 1A-B). Furthermore, neutrophil activation and NETs correlated significantly with each other (Fig. 1C). Next, we analyzed the data according to disease subtype and disease duration. We found a significant increase in neutrophil activation and a trend toward an increase in NETs in diffuse cutaneous SSc patients compared to limited cutaneous SSc patients (Fig. 2A-B). We also observed that the increases in neutrophil activation and NETs were more likely to occur earlier in the disease as both had a significant negative correlation with disease duration (Fig. 2C-D). These data suggest that neutrophils undergo activation and NET release that is most likely to be found in diffuse cutaneous SSc and earlier after disease onset.

### Association of neutrophil activation and NETs with vascular complications of SSc

**Fig. 3:**
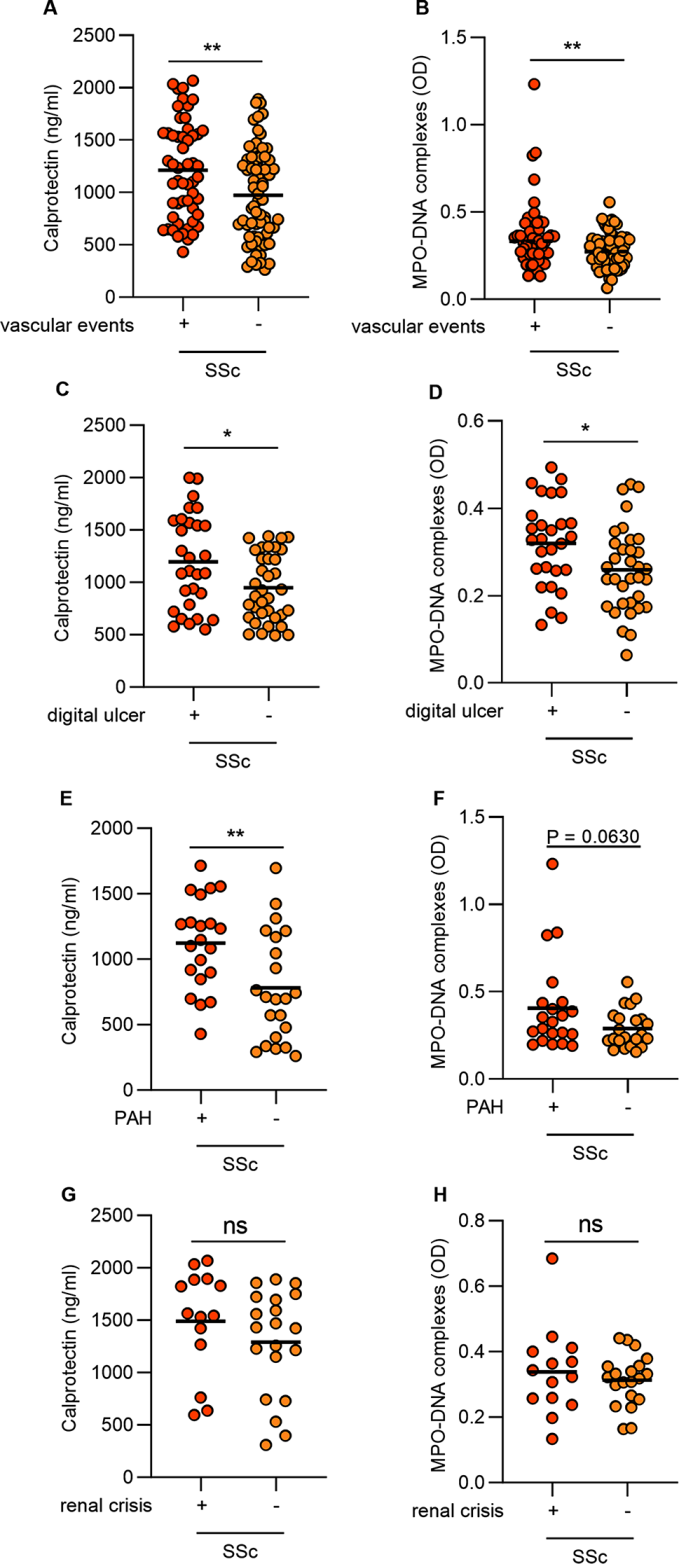
Increased neutrophil activation and NETs associate in SSc patients with vascular complications. (**A-B**) Levels of calprotectin (S100A8/A9) and MPO-DNA complexes were analyzed by ELISA in plasma samples of SSc patients with vascular complications (*n* = 54) and SSc patients without vascular complications (*n* = 71). Patients were grouped according to their clinical type of vascular complication. Levels of calprotectin (S100A8/A9) and MPO-DNA complexes in SSc patients with (**C-D**) digital ulcer, (**E-F**) pulmonary artery hypertension, and (**G-H**) history of renal crisis respectively. Mean is presented as a horizontal line; *P < 0.05, **P < 0.01, ns = not significant by t-test

Since neutrophils and NETs are key drivers of various vasculopathies [31], we speculated that they might also have a critical role in the vascular complications associated with SSc. Indeed, we found a significant increase in neutrophil activation and NETs in the plasma of SSc patients with vascular complications as compared to patients without vascular complications (Fig. 3A-B). We further investigated neutrophil activation and NETs according to the type of vascular complication. For both digital ulcers and PAH, neutrophil activation and NET levels were increased compared to matched patients without these features (Fig. 3C-D and E-F, respectively). Interestingly, the increases in neutrophil activation and NETs in patients with digital ulcers were more likely to be observed in patients with active digital ulcers as compared to patients with a history of digital ulcers that were not currently active (Supplemental Fig. 1A-F). No significant differences were observed in patients with a history of scleroderma renal crisis compared to matched patients without that history (Fig. 3G-H). Though we observed no differences related to a history of renal crisis, it should be noted that specimens from patients experiencing active renal crises were not available for this work. Recruiting such individuals into future studies would also allow us to evaluate local NET expression in kidney biopsy tissues. Taken together, these data suggest that the association of increased neutrophil activation and NETs with SSc vascular complications is more evident in the setting of active disease.

### NETs correlate with vascular injury in SSc

**Fig. 4:**
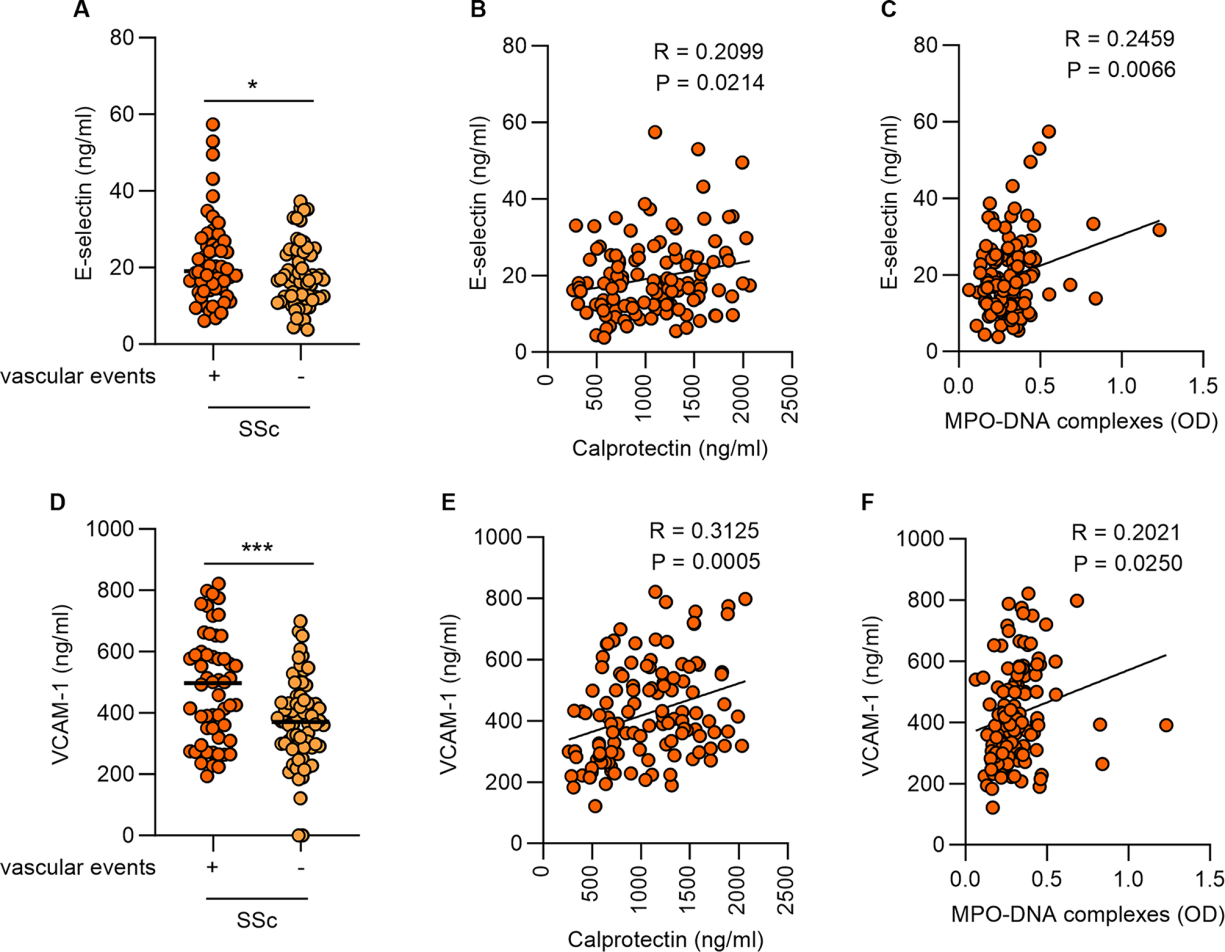
Neutrophil activation and NETs correlate with vascular injury in SSc patients. (**A**) E-selectin was analyzed by ELISA in plasma samples of SSc patients with vascular complications (*n* = 54) and SSc patients without vascular complications (*n* = 71). (**B**) Correlation between E-selectin and calprotectin (S100A8/A9). (**C**) Correlation between E-selectin and and MPO-DNA complexes. (**D**) VCAM-1 was analyzed by ELISA in plasma samples of SSc patients with vascular complications (*n* = 54) and SSc patients without vascular complications (*n* = 71). (**E**) Correlation between VCAM-1 and calprotectin (S100A8/A9). (**F**) Correlation between VCAM-1 and MPO-DNA complexes. Mean is presented as a horizontal line; *P < 0.05, ***P < 0.001 by t-test, and Pearson correlation was computed for B, C, E, and F

Vascular injury is an early event and a prominent hallmark of SSc. Given the observed differences in neutrophil activation and NETs in SSc with vascular complications, we next assessed vascular injury markers and their correlation with neutrophil activation and NETs. As expected, we found that SSc patients with vascular complications had higher soluble E-selectin compared to SSc patients without vascular complications (Fig. 4A). Furthermore, E-selectin correlated with neutrophil activation and NETs (Fig. 4B-C). Similarly, we observed higher soluble vascular cell adhesion molecule-1 (VCAM-1) in SSc patients with vascular complications compared to SSc patients without vascular complications (Fig. 4D). Like E-selectin, VCAM-1 was also significantly correlated with neutrophil activation and NETs (Fig. 4E-F). We also tested correlations between NET and vascular injury markers in only those patients with vascular complications, which trends similar to those observed for the whole cohort (Supplemental Fig. 2).

### The synthetic prostacyclin epoprostenol boosts SSc neutrophil cAMP and inhibits NET release

**Fig. 5:**
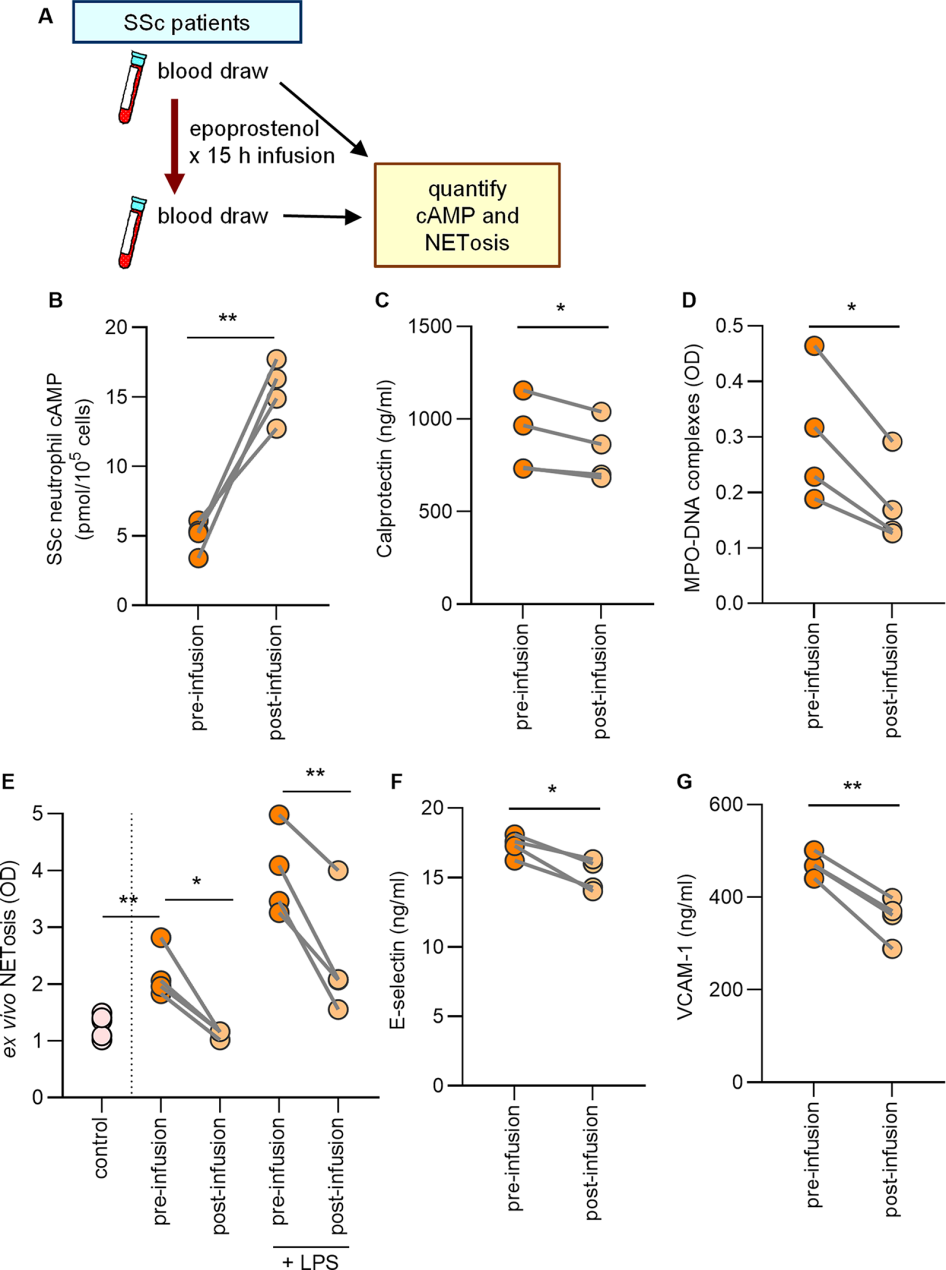
Prostacyclin epoprostenol boosts neutrophil cAMP and reduces ex vivo NETosis and vascular injury in SSc patients. (**A**) Four SSc patients with digital ischemia were infused with epoprostenol (0.45 mg in 150 ml 0.9% sodium chloride administered continuously over 30 h). One of these patients also had mild, stable PAH. Blood was collected the week before the infusion and 15 h after the start of the infusion (**B**) Levels of cAMP were assessed in neutrophils isolated from participants. (**C**) Neutrophil activation was assessed by measuring calprotectin (S100A8/A9). (**D**) NET levels in plasma were assessed by measuring MPO-DNA complexes. (**E**) Neutrophils were isolated from participants and cultured for 3 h, and some samples were additionally stimulated with lipopolysaccharides (LPS). NETs release was quantified by measuring extracellular DNA, and data are presented as fold-change in activation relative to unstimulated control neutrophils. Vascular injury was assessed by measuring (**F**) E-selectin and (**G**) VCAM-1 by ELISA in plasma samples. For all panels, the values of some data points overlap, *P < 0.05, **P < 0.01 by t-test

Prostacyclin mediates vasodilation by leveraging the second messenger cAMP. The receptor for prostacyclin, IP, is found on various cell types including neutrophils [32]. We previously showed that boosting neutrophil cAMP can modulate NET release in models of other rheumatic diseases such as lupus and APS [28, 29]. Thus, we speculated that synthetic prostacyclin analogs might be beneficial for protecting against SSc vasculopathy not only through their vasodilatory actions, but also through cAMP-mediated inhibition of NET release. We consented four SSc patients who were slated to receive infusions of the synthetic prostacyclin analog epoprostenol (0.45 mg in 150 ml 0.9% sodium chloride administered continuously over 30 h) for treating active digital ischemia (Fig. 5A). The main disease features, demographics, and concurrent drug treatments are detailed in Table 2. We sampled blood for isolation of neutrophils and plasma the week before starting the epoprostenol infusion and again 15 h after starting the epoprostenol infusion (halfway through the complete infusion protocol) (Fig. 5A). Compared to pre-epoprostenol samples, there was a significant increase in neutrophil cAMP after epoprostenol infusion (Fig. 5B). At the same time, there was a decrease in in vivo neutrophil activation and NET release as determined by measurement of calprotectin and MPO-DNA complexes, respectively (Fig. 5C-D). Similarly, there was a reduction in ex vivo NET release, whether spontaneous or stimulated by lipopolysaccharide (LPS) (Fig. 5E). Consistent with these observations, there was a reduction in the vascular injury markers E-selectin and VCAM-1 (Fig. 5F-G). It is worth mentioning that these patients were also using other medications (Table 2), such as sildenafil, a phosphodiesterase 5 inhibitor and vasodilator; however, the doses of concurrent medications were not changed between the first blood draw and the epoprostenol infusion. It is therefore likely that the experimental findings can be attributed to epoprostenol rather than the concurrent medications. These data demonstrate that epoprostenol altered SSc neutrophil activity in vivo, resulting in neutrophils less prone to NET release.

**Table 2:**
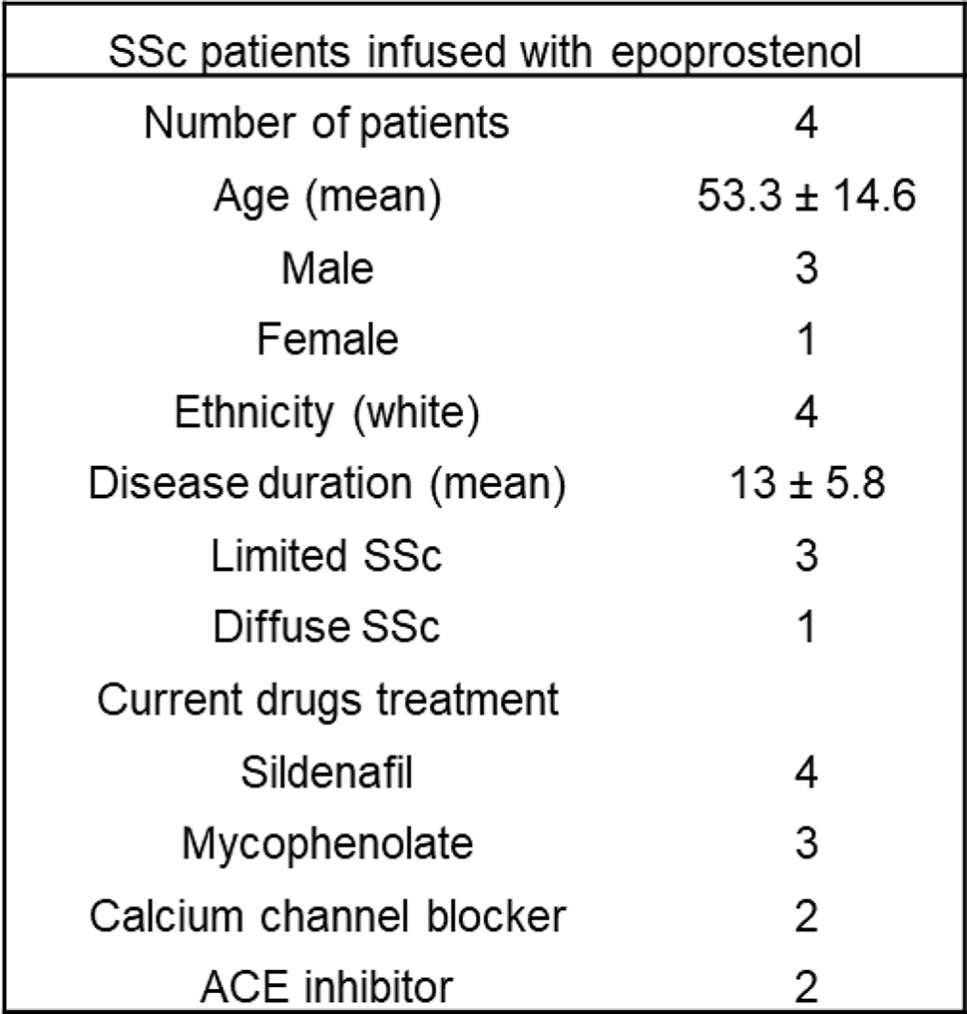
Main features and demographics for SSc patients infused with epoprostenol

## Discussion

SSc is a heterogeneous autoimmune fibrosing disease that jeopardizes patient survival and continues to present major challenges for physicians. It presents clinically with structural and functional dysfunction of the vasculature, autoimmunity, and widespread skin and organ fibrosis. Vascular injury is an early event that marks the initiation of SSc pathogenesis [1, 2], and many of the most devastating consequences of SSc are the result of a deranged vasculature. Neutrophils and their thrombo-inflammatory byproducts NETs have been revealed as drivers of diverse vasculopathies and highlighted as integral to the pathogenesis of various autoimmune and inflammatory diseases [10–15]. Yet, their role in SSc has not been comprehensively investigated. To date, only a few studies have assessed neutrophil hyperactivity and NET release in SSc [33–36]. In the current study, we investigated the role of neutrophils and NETs in the vascular complications of SSc. Here, we report an association between increased neutrophil activation and NETs in SSc patients and vascular complications. We further show that a synthetic prostacyclin analog inhibits NET release, potentially providing further value in combatting SSc vasculopathy.

We found that increased neutrophil activation and NETs were more likely to be associated with diffuse cutaneous SSc and shorter disease duration (Fig. 2). We notably observed increased neutrophil activation and NETs in SSc patients with vascular complications (Fig. 3). This pattern was most prominent in patients with ongoing active complications, such as those with active digital ulcers and PAH, as compared to patients without active complications, such as those with a history of digital ulcers or renal crisis (Fig. 3 and Supplemental Fig. 1). Our data build upon a previous study that examined a small cohort of SSc patients and found that NET release from SSc neutrophils appears to be linked with vascular complications and early disease duration [34]. We have extended these findings by also linking NETs to diffuse cutaneous SSc more so than limited cutaneous SSc (Fig. 2A-B). Notably, only eight individuals with diffuse cutaneous SSc were included in the prior study. A potential clinical implication of our findings is that circulating NETs may serve as a biomarker for identifying the subset of patients with an increased risk for current and future SSc vasculopathy. Such a marker would positively impact clinical practice and, likely, patient outcomes.

We can also now report that the synthetic prostacyclin epoprostenol has the potential to protect against SSc vasculopathy not only via vasodilation, but also by cAMP-mediated inhibition of NET release. We speculate that by increasing cAMP in SSc neutrophils, epoprostenol decreased neutrophil activation and NET release, plasma NET levels, and markers of vascular injury. These results are likely applicable to other prostacyclin analogs beyond epoprostenol although that question awaits further study. These results are consistent with previous reports demonstrating the inhibitory effect of prostacyclin on neutrophil function [37] and adhesion on vascular endothelial cells [38]. Interestingly, another member of the prostaglandin family PGE_2_ has also been shown to inhibit NET release by modulating the cAMP-PKA pathway through EP_2_ and EP_4_ Gαs-coupled receptors [39]. These results are also in agreement with our previous work demonstrating the importance of the cAMP pathway in modulating neutrophil hyperactivity in rheumatic diseases such as lupus and APS [28, 29].

Vascular injury is an early event in SSc and occurs in virtually all organs. It marks the initiation of SSc pathogenesis and triggers a self-fueling process that ends in tissue fibrosis and, ultimately, disrupts the architecture of the affected tissues. The contribution of NETs to vascular abnormalities, including endothelial dysfunction, has been previously reported in various vascular diseases [40]. In this study, we report elevated levels of vascular injury markers such as E-selectin and VCAM-1 (Fig. 4) in SSc patients, especially those with vascular complications, which agrees with previous studies [41, 42]. Notably, these markers correlated significantly with increased neutrophil activation and NETs. These soluble adhesion molecules released by endothelial cells may reflect the ongoing activation state and dysregulation of the endothelium [43]. These data may, therefore, suggest a crucial role for NETs in inducing SSc endothelial dysfunction. At the same time, an activated endothelium might trigger neutrophil activation and NET release [44], thereby setting up a vicious cycle. Importantly, the vascular injury was significantly reduced upon administration of a synthetic prostacyclin analog (epoprostenol) in SSc patients, which agrees with previous observations in SSc patients infused with the prostacyclin analog iloprost [45].

We postulate that the vascular protective effects of prostacyclin analogs are likely due to their combined action on multiple cell types. They mediate vasodilation and counter thrombosis by inhibiting vascular smooth muscle contraction and platelet aggregation [23, 27]. Likewise, they seem to modulate neutrophil function by reducing neutrophil hyperactivity and NETs, as we demonstrated in this study. Prostacyclin has also been shown to provide numerous endothelial protective effects [46–48]. One recent study demonstrated the ability of iloprost to stabilize adherens junctions and reduce endothelial-to-mesenchymal transition [49], while another suggested that the protective effects of iloprost could be attributed to downregulating CXCL10 release from endothelial cells and fibroblasts [50], together providing a mechanistic basis for the use of prostacyclin analogs in treating SSc patients with Raynaud’s phenomenon and digital ulcerations. These multifactorial actions likely work together to amplify the effects of prostacyclins, thus providing added protective effects in SSc.

A limitation of this study is the lack of analysis of other complications that could potentially be associated with NETs such as macrovascular complications, peripheral vascular disease (PVD), telangiectasias, and gastric antral vascular ectasia (GAVE). Furthermore, beyond our demonstrated connection between NETs and vascular complications in SSc, NETs have also been shown to promote fibroblast differentiation into myofibroblasts. Further study is needed to investigate their potential role in the fibrosis associated with SSc. Another drawback is the small sample size of the observational prostacyclin analog (epoprostenol) treatment study. Extending similar testing to the many randomized SSc clinical trials that deployed such agents could be an interesting and important future direction.

## Conclusions

In summary, our data demonstrate an association between NETs and vascular complications in SSc. Most likely, neutrophils from these patients have a reduced threshold for NET release, which portends an increased risk for current and future SSc vasculopathy. This state of increased neutrophil activity and NETs may identify patients at risk for vascular complications, thus offering potential clinical value as a biomarker to monitor the disease at an early stage and thereby improve outcomes. Our data also demonstrate that synthetic prostacyclin analogs reduce neutrophil hyperactivity and NET release in SSc patients, potentially providing additional therapeutic benefits for NET-associated SSc vasculopathy. Given that current prostacyclin analogs are challenging to administer, developing agents with longer half-lives that are amenable to oral administration should be considered to broaden therapeutic utility. Finally, the cascade of events leading to excessive NET release and the role of associated factors and mediators in SSc is still not clearly understood. Thus, future studies that systematically dissect the roles of neutrophils and NETs in this disease seem warranted.

### Electronic supplementary material

Below is the link to the electronic supplementary material.

Supplementary Material 1

## Data Availability

All primary data that support the findings of this study are available from the corresponding author upon reasonable request.

## Abbreviations

NETs: Neutrophil extracellular traps
SSc: Systemic sclerosis
HC: Healthy controls
PAH: Pulmonary artery hypertension
APS: Antiphospholipid syndrome
cAMP: Cyclic adenosine monophosphate
LPS: Lipopolysaccharides
