# Supplementary material for: Elevated neutrophil extracellular traps in systemic sclerosis-associated vasculopathy and suppression by a synthetic prostacyclin analog
Source: Arthritis Res Ther. 2024 Jul 25;26:139. doi: 10.1186/s13075-024-03379-6 (PMC11270934; doi:10.1186/s13075-024-03379-6)
Supplement: Supplementary file 1 — Supplementary Material 1 [file 13075_2024_3379_MOESM1_ESM.docx]

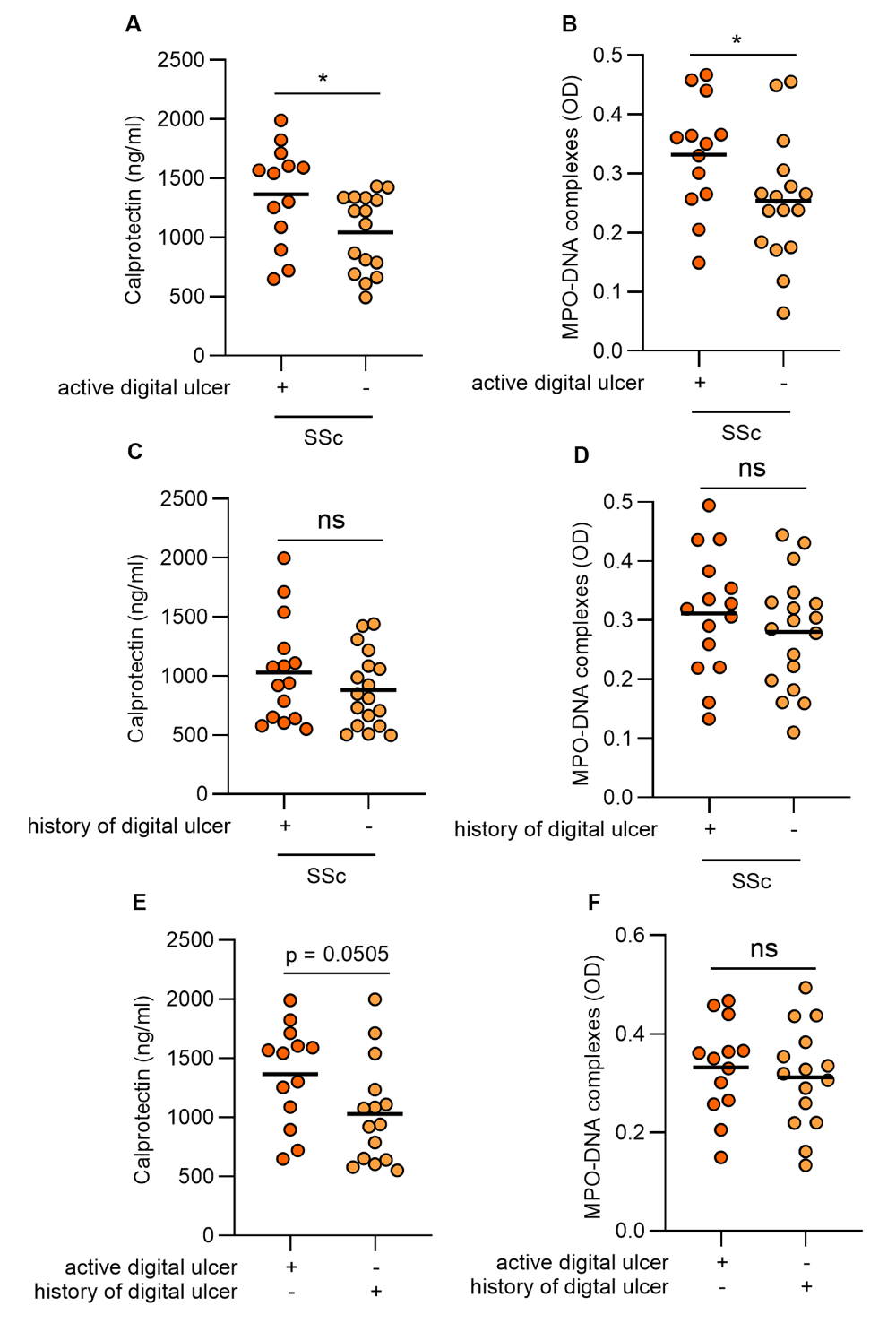


**Supplemental Figure 1: Increased neutrophil activation and NETs in SSc patients with digital ulcers are more apparent in the active disease state.** SSc patients with digital ulcers were grouped according to the disease state (active digital ulcers vs. history of digital ulcers). Levels of calprotectin (S100A8/A9) and MPO-DNA complexes in SSc patients with (**A-B**) active digital ulcers or with (**C-D**) a history of digital ulcers compared to matched patients without any history of digital ulcers. (**E-F**) Levels of calprotectin (S100A8/A9) and MPO-DNA complexes in SSc patients with active digital ulcers were compared to SSc patients with a history of digital ulcers. Mean is presented as a horizontal line; **P* < 0.05, ns = not significant by t-test.


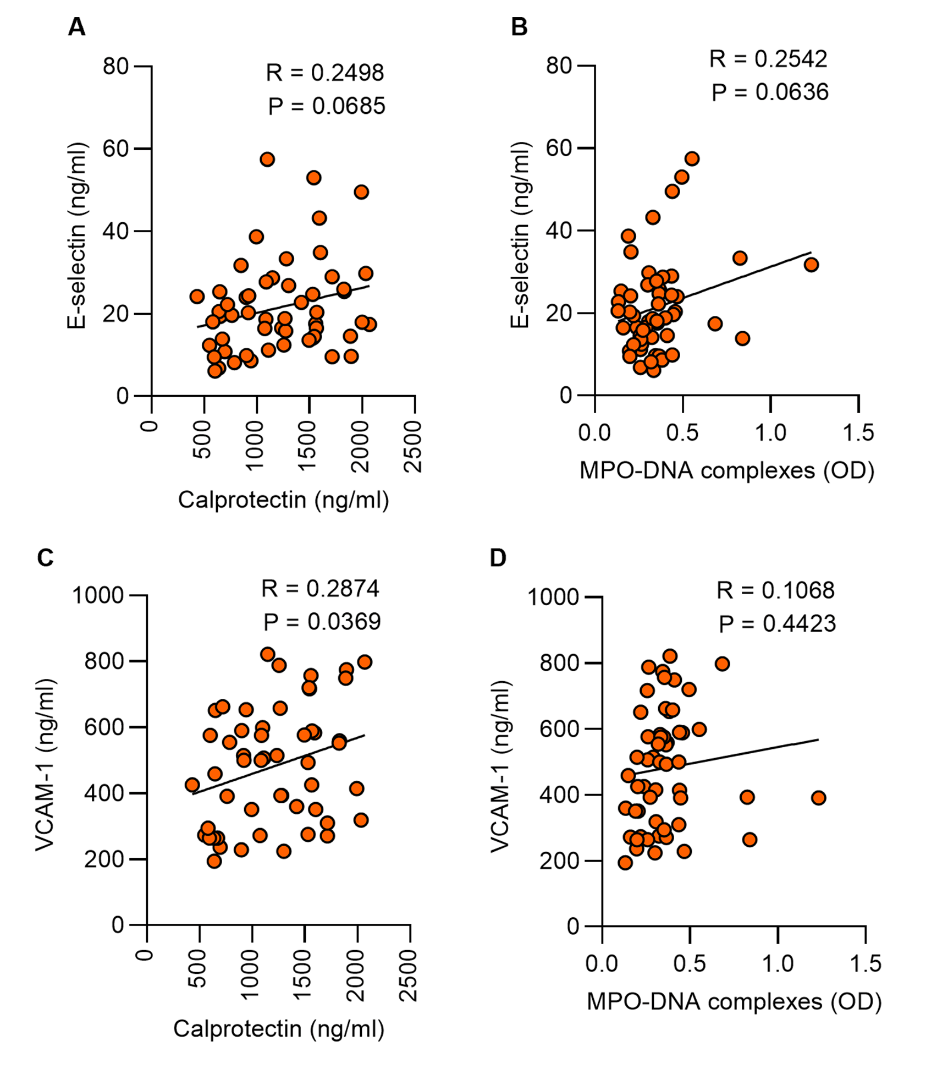


**Supplemental Figure 2: Correlation of neutrophil activation and NETs with vascular injury in SSc patients with vascular complications.** Vascular injury markers from SSc patients with vascular complications were analyzed for their correlations with neutrophil activation and NETs. (**A**) Correlation between E-selectin and calprotectin (S100A8/A9). (**B**) Correlation between E-selectin and and MPO-DNA complexes. (**C**) Correlation between VCAM-1 and calprotectin (S100A8/A9). (**D**) Correlation between VCAM-1 and MPO-DNA complexes. Pearson correlation was computed for all panels.
